# Factors associated with tuberculosis among patients attending a treatment centre in Zaria, North-west Nigeria, 2010

**DOI:** 10.11694/pamj.supp.2014.18.1.4189

**Published:** 2014-07-21

**Authors:** Ndadilnasiya Endie Waziri, Simeon Cadmus, Patrick Nguku, Olufunmilayo Fawole, Olajide Adewale Owolodun, Hyelshilni Waziri, Luka Ibrahim, Oladayo Biya, Saheed Gidado, Samuel Badung, Peterside Kumbish, Peter Nsubuga

**Affiliations:** 1 Nigeria Field Epidemiology and Laboratory Training Program, Nigeria; 2 Department of Veterinary Public Health, University of Ibadan, Nigeria; 3 Department of Epidemiology, Medical Statistics and Environmental Health, University of Ibadan, Nigeria; 4 National Veterinary Research Institute, Vom, Nigeria; 5 Global Public Health Solutions, Decatur GA, USA

**Keywords:** Tuberculosis, *mycobacterium tuberculosis* complex, polymerase chain reaction, adjusted odds ratio

## Abstract

**Introduction:**

Tuberculosis remains a global public health problem. In 2011, tuberculosis incidence was 133 per 100,000 in Nigeria. In Nigeria, little is known about the factors associated with tuberculosis, especially in the northern part and only few studies have characterized the *Mycobacterium species* that cause tuberculosis infection in humans. This study determined factors associated with tuberculosis and identified *Mycobacterium species* causing human tuberculosis in North-West, Nigeria.

**Methods:**

We conducted a hospital based case control study between April and July 2010 in Zaria. Cases were newly diagnosed sputum smear-positive tuberculosis patients >15 years while controls were patients >15 years attending the hospital for other reasons but were negative for tuber-culosis. We used a structured questionnaire to obtain information on demographics, knowledge of transmission of tuberculosis, and exposure to some factors. We preformed descriptive, bivariate and backward elimination logistic regression. Sputa from cases were analyzed by multiplex polymerase chain reaction (PCR) based on genomic regions of difference.

**Results:**

The mean ages of the cases and controls were 36, standard deviation (SD) 9.0 and 36, SD 9.7 respectively. Only 10 (9.8%) and nine (8.8%) of cases and controls respectively had a good knowledge of the transmission of tuberculosis. Contact with a tuberculosis patient (adjusted odds ratio (AOR) 12.3, 95% confidence interval (CI) 5.2-28.8), consumption of unpasteurized milk (AOR 6.4, CI 2.4-17.2), keeping pets (AOR 5.6, CI 2.3-13.7), associating closely with cattle (AOR 5.6, CI 1.3-6.8), and overcrowding (AOR 4.8, CI 1.8-13.1) were significantly associated with tuberculosis. Of the 102 sputa analyzed, 91 (89%) were *M. tuberculosis*, 8 (7.8%) were *M africanum*.

**Conclusion:**

We identified possible opportunities for intervention to limit the spread of tuberculosis. We recommend that the Nigeria tuberculosis control program consider some of these factors as a way to mitigate the spread of tuberculosis in Nigeria.

## Introduction

Tuberculosis is one of the most widespread infectious diseases and is the second leading cause of death from an infectious disease worldwide. In 2011, there were an estimated 8.5-9.2 million cases of tuberculosis and 1.2-1.5 million deaths from the disease globally. Most of the estimated number of cases occurred in Asia (59%) and Africa (26%). Despite the availability of efficacious treatment for decades, tuberculosis remains a major global health problem especially in resource poor nations and is threatening to re-emerge in developed nations as well [1–3]. The increase in population density, poverty, demographic changes, immigration, and the presence of Human Immunodeficiency Virus (HIV) are some of the factors responsible for this increase [4]. Tuberculosis is a major opportunistic infection in HIV-infected persons, the two forming a lethal combination and each speeding the other's progress [5]. Although *M. tuberculosis* is the most common cause of human tuberculosis, other species of the *Mycobacterium tuberculosis* complex (MTBC) are increasingly being recognised as a cause of human infection [6]. An important health concern is the zoonotic transmission of some strains of mycobacteria from animals to humans and vice versa. Of particular importance is the transmission of zoonotic tuberculosis to humans directly from cattle and through consumption of unpasteurized milk, and *M. bovis* bacille Calmette-Guérin (BCG) infection of immune-compromised individuals [7, 8]. *Mycobacterium tuberculosis*, the predominant cause of human tuberculosis, is thought to be the most successful human bacterial pathogen and the most poorly understood [9]. In developing countries, most laboratories rely on sputum microscopy Zeihl-Neilsen (ZN) acid-fast test, as a cornerstone for diagnosis of tuberculosis in resource limited settings. However, the ZN test does not fully identify species of MTBC isolates. Since most laboratories do not fully identify MTBC isolates, the true cause of tuberculosis in these patients, its source and associated factors often remain undiscovered. Also, it is more difficult to diagnose tuberculosis by sputum microscopy in HIV-infected persons [10].

In Nigeria, tuberculosis incidence was estimated to be 133 cases per 100,000 with a tuberculosis-HIV co-infection rate of 25% in 2011 [1]. Studies investigating the factors associated with tuberculosis have been conducted in a variety of settings, but very few in Africa [11]. There is also little or no surveillance in Nigeria for the control of zoonotic tuberculosis and pasteurization of milk is not enforced; meanwhile, pastoral communities live in close contact with their cattle. These cultural practices could facilitate transmission of tuberculosis between cattle and humans [12]. Little is known about the factors associated with tuberculosis in Nigeria especially in the northern region of the country which has the highest concentration of cattle and only few studies have characterized the *Mycobacterium species* that cause tuberculosis infection in humans including the possibility of zoonotic species [12]. We conducted a study to identify factors associated with tuberculosis and determine the species of MTBC causing tuberculosis in Zaria, North-West, Nigeria. This will enable TB program managers to give advice and implement effective control measures in this environment.

## Methods

We undertook a hospital based case control study in a Directly Observed Treatment Short course (DOTS) centre in Zaria, north-west Nigeria in 2010. The outpatient unit sees approximately 200 patients per day, two times a week. Patients can be admitted directly to an onsite ward for hospital care. Using the sample size calculation module of Epi. Info 3.3.1 software (U.S. Centers for Disease Control and Prevention), we estimated that 112 cases and 112 controls should be recruited to achieve a power of 80% to detect and odds ratio of 3 at 5% significance if 10% of the general population are exposed to the factors of interest such as previous contact with a tuberculosis patient. Cases were defined as newly detected sputum smear positive pulmonary tuberculosis patients >15 years diagnosed between 1st April 2010 and 3rd July 2010. Pulmonary tuberculosis was confirmed by two consecutive sputum smears positive for acid-fast bacilli. Controls were defined as patients >15 years attending the hospital for other reasons but were negative for tuberculosis both clinically and microbiologically. All the 102 pulmonary tuberculosis patients diagnosed during the study period were recruited consecutively into the study. Controls were systematically selected by picking every third non-tuberculosis patient seen at the hospital during the same period until the desired number was attained.

**Data collection and analysis:** We administered a structured questionnaire to the 102 cases and 102 controls to obtain information on demographics, knowledge of transmission of tuberculosis, and exposure to potential host-related and environment-related factors. These factors included previous clinical history, HIV status, general living condition, education level, type of employment, eating habits, and history of keeping either dogs or cats. The presence of BCG scar on the deltoid region of the arm was checked. We graded knowledge of transmission of tuberculosis on a scale of 0 to 3 representing no knowledge which was given a score of 0; poor knowledge a score of 1; fair knowledge, a score of 2; and good knowledge (a score of 3) of transmission of tuberculosis. We defined overcrowding as having more than three persons living in a room. Data were entered and analyzed using Epi-info version 3.3.1 statistical software and Microsoft Excel 2007. Descriptive statistics and bivariate analyses were conducted. Adjusted Odds Ratios and 95% confidence intervals were estimated for variables significant at p < 0.05 level in the bivariate analyses using the backward elimination logistic regression.

**Sputum collection and preparation:** Patients provided an on the spot sputum specimen on the first day of presentation at the hospital and were given sterile containers for next day early morning sputum as a second sample. A third sputum sample was collected when patients returned to the hospital the next morning. Sputum specimens were decontaminated as previously described by Abbadi et al., [13]. Direct smears were prepared from each of the specimens and Ziehl Neelsen (ZN) staining was performed on all smears. One sputum specimen with the highest ZN count’ > with the highest ZN count from each of the cases was processed for molecular typing.

**DNA extraction:** DNA from sputa of the cases was extracted and purified as described previously by Haurd et al. [7]. Sputum specimens were digested with proteinase K and Lysozyme before extracting DNA.

### Polymerase Chain Reaction (PCR) amplification and analysis of target DNA

**a). Multiple:** xMultiplex PCR was performed using two primer sets; INS1 & INS2; JB21 & JB22, and the procedure described previously by Figueiredo et al., [14]. Specific primers INS1 and INS2 identify bacteria as MTC members whereas JB21 and JB22 distinguish *M. bovis* isolates from other members of this complex. Amplification was carried out in a GeneAmp PCR System 9600 (Applied Biosystems^®^, Singapore) and PCR products analyzed by agarose gel electrophoresis. DNA bands were visualized and captured by ChemiGenius BioImaging System (Syngene^®^, Cambridge, UK).

**b). Genomic Region of Difference:** For speciation of the MTC, deletion analysis was carried out as previously described by Warren et al., [15] using four primers namely RD1, RD4, RD9, RD12. Each PCR reaction contained 1µl DNA template, 5 µl Q-buffer (Qiagen^®^, Hilden, Germany), 2.5 µl 10x PCR buffer (Qiagen^®^, Hilden, Germany), 2 µl of 25 mM MgCl2, 4 µl 10 mM dNTPs, 0.5 µl of each primer (50pmol/µl) (Inqaba^®^, Pretoria, South Africa), 0.25 µl HotStar Taq DNA polymerase (Qiagen^®,^ Hilden, Germany) and was made up to 25µl with nuclease free water (Promega^®^, Madison, USA). Amplicons were electrophoresed through 2.0% Agarose supplemented with 50µg ethidium bromide (Promega^®^, Madison, USA), at 120V for 90 minutes. DNA bands were visualized and captured by ChemiGenius BioImaging System (Syngene^®^, Cambridge, UK).

**Ethical considerations:** We obtained ethical clearance for the study from the Kaduna state ethical review committee. In addition, informed consent was given by all study participants.

## Results

Demographic characteristics of study subjects: The mean age of the cases and controls was 36 with a standard deviation (SD) of 9.0 and 9.7 years respectively. An equal number of male respondents 72 (70.6%) were recruited among both cases and controls. Majority of the cases (83 (81.4%)) and controls (80 (78.4%)) were married (Table 1).

**Table 1:** Demographic Characteristics of Pulmonary Tuberculosis Cases and Controls in Zaria, 2010

Variable	Cases No. (%)	Controls (%)
**Age (years)**		
Mean (std dev)	36·2 (±9)	35·8(±9·7)
**Gender**		
Male	72 (70·6)	72 (70·6)
Female	30 (29·4)	30 (29·4)
**Marital Status**		
Single	19 (18·6)	22 (21·6)
Married	83 (81·4)	80 (78·4)
**Occupation**		
Unemployed	3 (2·9)	3 (2·9)
Informal	65 (63·7)	58 (56·9)
Formal	28 (27·5)	31(30·4)
Student	6 (5·9)	10 (9·8)
**Education level**		
Below Secondary school	43 (42·2)	35 (34·3)
At least secondary school	59 (57·8)	67 (65·7)
**HIV positive**	21 (20·6)	20 (19·6)

Knowledge of transmission of tuberculosis: Sixty one (59.8%) of the cases and 55 (53.9%) of the controls said tuberculosis was spread by air while a few, seven (6.9%) of the cases and two (2%) of the controls thought it was spread by insects. On a score scale of 0-3, representing no knowledge, poor knowledge, fair knowledge, and good knowledge of the transmission of tuberculosis, only 10 (9.8%) and nine (8.8%) of cases and controls respectively had a good knowledge of the transmission of tuberculosis. Rating for knowledge of at least one mode of transmission of tuberculosis showed that majority 72 (70.6%) and 60 (58.8%) of cases and control respectively had knowledge of at least one mode of transmission of tuberculosis.

Factors associated with tuberculosis among respondents: Cases that had a history of contact with a known tuberculosis patient, consumption of unpasteurized milk and milk products, and associating closely with cattle were 10, 6, and 6 times more likely to have tuberculosis than controls. Keeping pets at home was associated with a two times higher likelihood of having tuberculosis (Table 2).

**Table 2:** Factors associated with Pulmonary Tuberculosis among Patients in Zaria, 2010

Variable	Cases No. (%)	Control No. (%)	OR	95% CI
Consumption of under-cooked meat	101 (99)	102 (100)	0	0
Consumption of unpasteurized milk & milk products	86 (84·3)	41 (39·2)	8.3	4·3-16.2
Having pets at home	61 (59·8)	44 (43·1)	2.0	1·1-3·4
Associate closely with cattle	65 (63·7)	29 (28·4)	4·4	2·5-8.0
Previous contact of TB patient	68 (66.7)	17 (16·7)	10.0	4.9-20.6
HIV positive	21 (20·6)	20 (19·6)	1·1	0·5-2·3
Married	83 (81·4)	80 (78·4)	1·2	0·6-2·4
> 3Persons per room	43 (42.2)	13 (12.8)	6.7	3.1-14.4
Taken BCG Vaccination	79 (77·5)	83 (81·4)	0·8	0.4-1·6

Logistic regression model for factors associated with tuberculosis among patients in Zaria, 2010: In a logistic regression containing marital status, HIV status, knowledge of transmission of tuberculosis, BCG status, consumption of unpasteurized milk and milk products, previous contact with a known tuberculosis patient, associating closely with cattle, keeping pets such as dogs and cats at home, and overcrowding (having more than three persons living in a room), consumption of unpasteurized milk and milk products, previous contact with a known tuberculosis patient, associating closely with cattle, keeping pets, and overcrowding were significantly associated with being a case of tuberculosis (Table 3).

**Table 3:** Logistic Regression Model for Factors associated with Pulmonary Tuberculosis among Patients in Zaria, 2010

Variables	Adjusted Odds Ratio	95% Confidence Interval
Have had contact with a TB patient	12.3	5.2-28.8
Consumption of unpasteurized milk and milk products	6·4	2·4-17.2
Keeping pets at home	5.6	2·3-13.7
Overcrowding	4.8	1·8-13.1
Associating with cattle	2.6	1·3-6·8

**Figure 1:**
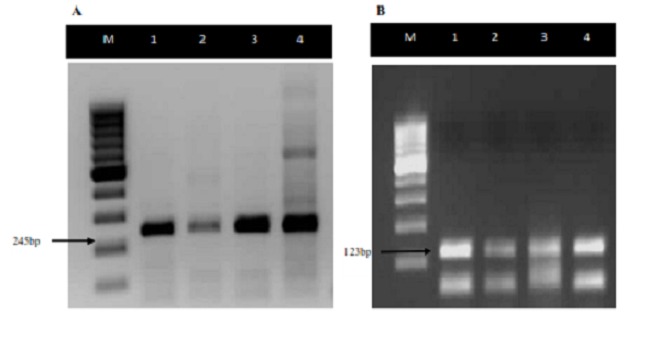
Agarose gel electrophoresis showing diagnosis of MTC by 2 PCR reactions to detect (A) the 245 bp fragment from IS6110 and (B) the 123 bp fragment from IS6110. For (A) - Lanes 1, 2, and 3 show *M. tuberculosis* positive specimens; Lane 4 shows a *M. tuberculosis* positive control with a 100bp molecular weight marker (Lane M). For B - Lanes 1,2 and 3 show the 123 bp *M. tuberculosis* fragment, and lane 4 shows MTB positive control

Detection of MTC in sputum of tuberculosis patients: In all the 102 smear positive sputum specimens tested by multiplex-PCR among the cases, multiplex-PCR successfully amplified the target region, 245-bp and 123-bp fragments typical and diagnostic for *Mycobacterium tuberculosis* complex. The 500-bp fragment specific for *M. bovis* was not amplified in any specimens (Figure 1). Based on on the confirmation of fragment sizes for the 4 regions of difference depicting presence or absence of specific region of difference, 91 (89.2%) of the sputa were identified as *M. tuberculosis* while 8 (7.84%) were *M africanum* and 3 (3%) were mixed infections of *M. tuberculosis* and *M. africanum*. In total, 94 and 11 *M. tuberculosis* and *M. africanum* were identified.

## Discussion

In this study, we found previous exposure to a tuberculosis patient, consumption of unpasteurized milk and milk products, associating closely with cattle, keeping of pets, and overcrowding were independent factors associated with tuberculosis among patients attending a DOTS center in Zaria in 2010. We also identified *M. tuberculosis* (89.2%) and *M. africanum* (10.8%) as species of MTBC causing human tuberculosis in Zaria, Nigeria. While the first three factors (consumption of unpasteurized milk and milk products, previous exposure to a tuberculosis patient, associating closely with cattle) have been previously documented as risk factors for tuberculosis [6, 11, 14, 16, 17], there are limited studies showing the association between keeping pets with the development of tuberculosis; hence, making our finding informative. In our study cases of tuberculosis were four times more likely to keep pets than controls. This is of great public health concern as it shows the possibility of zoonotic transmission. It is generally known that most pets are kept indoors and usually interact closely with members of the household especially children. Shrikrishna et al., [18], reported a case of human and canine pulmonary *M. bovis* (with an identical genotype) infection among two household members and their pet dog but could not ascertain if the transmission was from the pet to the humans or vice versa. However, since the scope of this study was limited to tuberculosis in humans, we cannot make conclusive statements on the role of pets in the epidemiology of tuberculosis and keeping pets would have been associated with another risk factor for tuberculosis that was not measured in this study.

Our study also shows that cases having more than three persons living in a room increased the likelihood of developing tuberculosis. Increased household size and overcrowding have been documented as a risk factor for tuberculosis from several other studies in a variety of settings [17–19]. This is an important finding considering that inhalation is the major route of transmission of tuberculosis. Adult crowding reflects the increased likelihood of coming into contact with infectious persons excreting the bacilli in crowded environments, but is also a marker of socioeconomic status [20, 21]. Poverty has been documented as one of the greatest risk factor for tuberculosis [17]. Although tuberculosis-HIV co-infection was high (20.6%), it is less than the 25% reported by WHO for Nigeria [1]. In The Gambia, a tuberculosis prevalence of 8% was reported in sputum smear positive cases [11]. We did not find a difference in HIV prevalence between cases and controls. Our findings could indicate a possible decline in tuberculosis-HIV co-infection rates. We found *M. tuberculosis* and *M. africanum* as species of MTBC causing human tuberculosis in North-western Nigeria. The finding of *M. tuberculosis* as the major cause of TB is in agreement with reports of a study done in South-Western Nigeria identified where *M. Tuberculosis* accounted for 85% of all tuberculosis cases [14].

The finding of *M. africanum* which has also been isolated from tissues of slaughtered animals and milk from pastoral cattle in Nigeria, suggests possible zoonotic transmission of this organism previously thought to be limited to humans [4, 14, 22, 23]. Our study reaffirms that the possibility of transmission of zoonotic tuberculosis and other milk-borne pathogens through the consumption of contaminated milk and milk products should not be underestimated. The absence of *Mycobacterium bovis* in this study does not rule out possibility of zoonotic transmission of bovine tuberculosis in this region, as there are various cultural practices such as herdsmen living with their cattle and consumption of unpasteurized milk and milk products, which could facilitate the transmission of *M. bovis* between cattle and humans. A prevalence of 5% of *M. bovis* associated pulmonary tuberculosis was reported among humans in the southern part of Nigeria [14]. In countries with relatively high prevalence of bovine tuberculosis in cattle, abattoir and farm workers are the groups most exposed with majority of infections presenting as extra-pulmonary tuberculosis [4]. Generalizing our findings beyond the study population is subject to at least two limitations. First of all, our study only looked at pulmonary tuberculosis patients and we analyzed sputa to detect pulmonary tuberculosis, extra pulmonary infections which are common with *M. bovis* in humans may have been missed. Finally, the people most at risk of infection with *M. bovis* (abattoir workers and livestock handlers) were not the primary target of this study.

## Conclusion

This study has identified possible opportunities for intervention to limit the spread of tuberculosis in northwest Nigeria. Consumption of unpasteurized milk and milk products was common and associated with tuberculosis, ways to ensure safe milk to the general populace is important. Since a history of contact with known tuberculosis patient was associated with tuberculosis, active tracing of tuberculosis case contacts should be considered to encourage early attendance at tuberculosis clinics and ways to avoid overcrowding should be encouraged. In addition, it is important to further study the role of pets in the epidemiology of tuberculosis in Nigeria. We recommend that the Nigeria tuberculosis control program consider some of these factors as a way to mitigate the spread of tuberculosis in Nigeria.
